# Bioavailability of Hesperidin and Its Aglycone Hesperetin—Compounds Found in Citrus Fruits as a Parameter Conditioning the Pro-Health Potential (Neuroprotective and Antidiabetic Activity)—Mini-Review

**DOI:** 10.3390/nu14132647

**Published:** 2022-06-26

**Authors:** Kamil Wdowiak, Jarosław Walkowiak, Robert Pietrzak, Aleksandra Bazan-Woźniak, Judyta Cielecka-Piontek

**Affiliations:** 1 Department of Pharmacognosy, Poznan University of Medical Sciences, Rokietnicka 3, 60-806 Poznan, Poland; kamil.wdowiak@student.ump.edu.pl; 2 Department of Pediatric Gastroenterology and Metabolic Diseases, Poznan University of Medical Sciences, Szpitalna 27/33, 60-572 Poznan, Poland; jarwalk@ump.edu.pl; 3 Faculty of Chemistry, Adam Mickiewicz University in Poznań, Uniwersytetu Poznańskiego 8, 61-614 Poznań, Poland; pietrob@amu.edu.pl (R.P.); aleksandra.bazan@amu.edu.pl (A.B.-W.)

**Keywords:** hesperidin, hesperetin, bioavailability, neuroprotection, antidiabetic

## Abstract

Hesperidin and hesperetin are polyphenols that can be found predominantly in citrus fruits. They possess a variety of pharmacological properties such as neuroprotective and antidiabetic activity. However, the bioavailability of these compounds is limited due to low solubility and restricts their use as pro-healthy agents. This paper described the limitations resulting from the low bioavailability of the presented compounds and gathered the methods aiming at its improvement. Moreover, this work reviewed studies providing pieces of evidence for neuroprotective and antidiabetic properties of hesperidin and hesperetin as well as providing a detailed look into the significance of reported modes of action in chronic diseases. On account of a well-documented pro-healthy activity, it is important to look for ways to overcome the problem of poor bioavailability.

## 1. Introduction

Hesperidin and hesperetin are both citrus flavonoids possessing a wide variety of biological activity. Hesperidin can be richly found in citrus fruits such as lemon (*Citrus Limon*), sweet oranges (*Citrus sinensis*), bitter orange (*Citrus aurantium*), citron (*Citrus medica*) [1], clementines (*Citrus clementina*), and mandarins (*Citrus reticulata*) [2]. Apart from citrus fruit, hesperidin can be found in *Menthae piperitae*, *Hypericum perforatum*, and *Salvie officinalis* [2]. Considering that hesperetin can be viewed as a metabolite of hesperidin, it is available in the same range of plant materials.

Interestingly, hesperidin can be easily isolated from the waste residues from citrus fruit processing, making it economically attractive [3,4]. In turn, hesperetin in the industry can be obtained by modifying hesperidin with the use of bacterial enzymes [5]. This makes the aglycone production process more complicated than that of the starting hesperidin and therefore the production costs arise.

**Figure 1:**
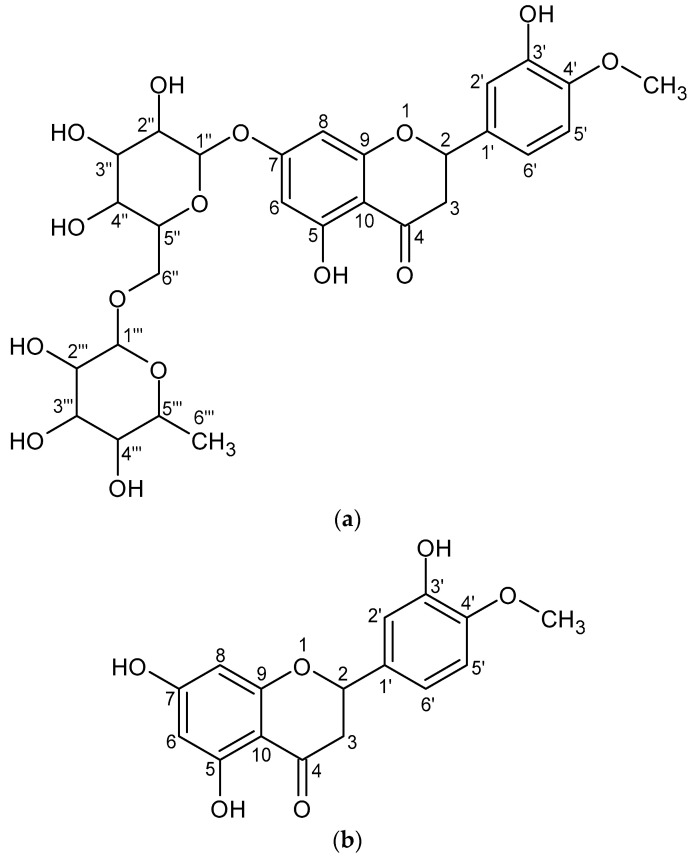
The chemical structures of hesperidin (**a**) and hesperetin (**b**). The structures were obtained via ACD/ChemSketch 2021.2.1.

Hesperidin is a polyphenolic plant compound from the flavonoid group; to be more specific, it belongs to flavanones. Flavonoids have a core structure of three-ring diphenyl propane (C_6_-C_3_-C_6_) and the skeleton that contains two benzene rings linked by a C_3_ moiety [6,7]. Flavanones, a subsection of flavonoids, are characterized by having a ketone group at the C_4_ atom and no double bond between the C_2_ and C_3_ atoms in the C ring [8]. Flavonoid glycosides are the major form of flavonoids in plant materials. The hydroxyl groups of flavonoids are involved in the formation of O-glycoside bonds. However, compounds that have a sugar residue attached to the flavonoid ring by a C-glycosidic bond can also be found [6]. When it comes to hesperidin (Figure 1a) from a chemical point of view, it is a flavanone glycoside and consists of an aglycone part—hesperetin (Figure 1b) (3′,5,7-trihydroxy-4′methoxyflavonone) and sugar moiety, which is rutinoside, i.e., a disaccharide composed of rhamnose and glucose, where glucose is attached to C_7_ of the hesperetin ring. The total molecular formula is C_28_H_34_O_15_ and the molecular weight is 610,57 Da [9].

Some of the benefits of the potential use of hesperidin are its safety profile, non-accumulative nature, and restricted side effects, even during pregnancy. In the study, aiming at assessing hesperidin safeness, it was administered at doses up to 5% in mice and no mutagenic, toxic, or carcinogenic effects were reported, even when given for a relatively long time [10]. Moreover, oral administration in humans caused minor adverse effects, only in 10% of patients [11]. However, there were some interactions reported of hesperidin with drugs such as vincristine and daunomycin, which should be considered [10].

This paper aimed to look into the bioavailability problem of hesperidin and hesperetin and to highlight the neuroprotective and antidiabetic potential of these compounds by reviewing modes of action and their importance in the development and progression of neurodegenerative diseases and diabetes mellitus.

In order to find relevant publications, a search was conducted in Google Scholar and Pubmed databases with the following keywords: “Hesperidin bioavailability improvement” and “hesperetin bioavailability improvement” for the search of bioavailability-enhancing techniques of the compounds. The papers regarding pure compounds and describing the effect on physicochemical properties were included. As far as biological studies are concerned, in search the phrases “hesperidin neuroprotective activity”, “hesperetin neuroprotective activity”, “hesperidin antidiabetic activity”, and “hesperetin antidiabetic activity” were applied. The studies concerning pure compounds (not extracts) were included. All articles published between 2012 and 2022 were considered for eligibility.

## 2. Bioavailability

Bioavailability is a key factor in observing the therapeutic effect of a given drug, especially taking into account oral administration, which is the most popular route of drug application in the daily therapy of various diseases [12]. Bioavailability is influenced by many factors, among which solubility and permeability through biological membranes play a significant role [13,14]. Solubility means the presence of a compound in the form of a free, unbonded molecule. The greater solubility is, the larger the quantity of molecules presented in a free form that enable the absorption of a compound. In turn, permeability means the ability to transport through biological membranes, i.e., the main barriers of the body that can limit the amount of absorbed substance [15]. For example, when administered orally, the walls of the gastrointestinal tract should be considered as the biological membrane. They constitute an obstacle to the availability of the drug but play a crucial physiological role. For example, they protect the body against toxins or pathogens from the external environment, ensuring survival. The penetration can occur by several transport routes, such as the passive or active pathway [14].

As far as the bioavailability of hesperidin and hesperetin is concerned, it can be described as rather complicated. It is estimated to be about 20% [16]. First of all, the poor solubility of these compounds leads to the release of small amounts of free molecules into the aqueous environment of the gastrointestinal tract, which translates into the penetration of modest quantities through biological membranes [16]. Moreover, hesperidin and hesperetin face an obstacle in the gastrointestinal tract countering their absorption, i.e., they are substrates of P-glycoprotein [17]. P-glycoprotein is a protein responsible for the efflux of substances, i.e., throwing out the absorbed substance outside the cell. This is a significant barrier to the bioavailability of many drugs [18,19,20]. Hence, despite penetration into the intestinal epithelial cells, these compounds can still be released back into the external environment. However, it has been reported that hesperidin and hesperetin may also act as inhibitors of P-glycoprotein, which have been shown in studies on cancer cells resistant to anticancer drugs. In these studies, it was observed that the anticancer drug hesperidin/hesperetin combination leads to an increase in the bioavailability of the anticancer drug and enhances the effectiveness of therapy, attributed to the ability to inhibit the action of P-glycoprotein, which is the main factor of multidrug resistance in cancer cells [21,22,23].

Additionally, hesperidin, unlike its aglycone, has poor membrane permeability and hence is said to be mainly absorbed via the paracellular pathway [24,25], meaning that the tight connection of intestinal cells may limit its absorption [17]. It is worth mentioning that studies indicate that hesperetin is able to cross the blood–brain barrier, which is an important factor considering its neuroprotective activity [26]. In other words, this compound is capable of reaching the site of disease in the case of neurodegenerative disorders and acting directly where pathological processes occur.

The microflora of the small intestine play an important role in the bioavailability of hesperidin, their task being to cut off the sugar moiety, which leads to the conversion to the aglycone form—hesperetin. This is due to the presence of the enzyme α-rhamnosidase secreted by *Bifidobacterium pseudocatenultum*, which releases the aglycone by detaching the sugar moiety—rutinoside [27]. Aglycone—hesperetin, which can also be described as an active form, after the modification can be absorbed by colonocytes and enter the systemic circulation, thus enabling further distribution to tissues [28]. Interestingly, hesperidin can modulate the gut microflora by promoting the growth of beneficial bacteria [29] and inhibiting the growth of pathological bacteria [30]. A detailed relationship between the microbiome and hesperidin was described by Mas-Capdevil et al. [28] as well as Stevens et al. [31].

Bearing in mind the important role of the intestinal microbiome and overall difficulties in oral bioavailability of hesperidin, it can be said that the observed effects when it comes to the pharmacological effect of hesperidin administration could be ascribed to its aglycone, hesperetin, which can be referred as an active form of hesperidin [32,33].

Taking into account the above factors, restricting the bioavailability of hesperidin and hesperetin, there is an urgent need to solve these difficulties in order to enhance their availability and enable the use of their pharmacological potential in the treatment of chronic diseases. So far, there have been several attempts to increase the solubility and permeability of these compounds. They are collected in Table 1.

## 3. Neuroprotective Potential of Hesperidin and Hesperetin

Neurodegenerative diseases are considered a major problem in the following years due to long life expectancy. Since these chronic conditions are age-related, the older society is, there is a greater likelihood that the occurrence of disorders connected to loss in neuronal function would appear to be troublesome for health services. Neurodegenerative diseases are characterized by two factors: (i) the vulnerability to disease of neurons in particular regions in the brain, and (ii) the progression of the disorder and its worsening in time. Some conditions are classified into this category of disorders such as Alzheimer’s disease, Parkinson’s disease, and Huntington’s disease [57]. Complexed pathology of neurodegenerative diseases makes many potential modes of action arise.

Hesperidin and hesperetin, owing to their antioxidant, anti-inflammatory, anti-amyloidogenic and anti-apoptotic activities, seem to have the potential to become promising neuroprotective agents. The studies indicating their neuroprotective potential are collected in Table 2.

### Importance of Hesperidin and Hesperetin Modes of Action in Neuroprotective Activity

The brain possesses a high demand for oxygen supply. It is said that it consumes approximately 20% of the total oxygen supply [83]. A considerable quantity of oxygen is converted to Reactive Oxygen Species (ROS), which can cause significant damage. Exposure of neurons to oxidative stress is a cause of their degeneration [84]. Oxidative stress is highlighted to be present in various neurodegenerative conditions [85].

Alzheimer’s disease (AD) is a progressive type of dementia and neurodegenerative disorder associated with the accumulation of intracellular tangles (hyperphosphorylated tau proteins) and extracellular β-amyloid plaques [86]. Oxidative stress is considered a major factor in the pathogenesis of AD and there can be different initiators causing its production [87]. In AD, β-amyloid is generated and it accumulates in neurons. When amyloid tangles are assembled in mitochondria, it leads to mitochondrial dysfunction. The energy metabolism occurs and since mitochondria are the main sources of ROS, there is excessive production of oxidative stress, which further damages the cellular structure, triggering apoptotic cell death, and therefore the loss of cell functions [88]. β-amyloid deposition causes glial cells activation, which promotes neuroinflammation. Various mediators of inflammation such as cyclooxygenase, chemokines, and cytokines are expressed [86]. Moreover, the myeloperoxidase is involved in ROS production by activated microglia [89]. There is a noticeable gathering of advanced glycation end products (AGEs) with age, which play an important role in ROS production. Moreover, there is strong evidence that AGE leads to ROS generation by reacting with the RAGE receptor [90]. It is often underlined that amyloid-induced oxidative stress plays a crucial role in Alzheimer’s disease genesis [91]. Not only does it cause mitochondrial dysfunction, but it also generates NMDA receptor overreaction. Abnormal NMDA receptor function is associated with calcium influx, induction of mitochondrial damage, and ROS production by increasing the mitochondrial calcium load [92]. Interestingly, oxidative damage promotes β-secretase activity, which in turn elevates β-amyloid1–42 levels [93]. It can be said that β-amyloid generates a vicious circle; it is a cause of ROS production, and dysfunction generated by ROS leads to an increase in amyloid levels. However, there are protective mechanisms that fight oxidative stress such as the enzymatic antioxidant system. It includes glutathione peroxidase (GPx), superoxide dismutase (SOD), and catalase (CAT). These enzymes take part in neutralizing ROS, thus protecting cells from damage [94]. Moreover, nuclear factor erythroid 2–related factor 2 (Nrf2) and its proteins—heme oxgenase-1 (HO-1) and quinone oxidoreductase 1 (NQO1) contribute to the essential antioxidant defense pathway [95]. The promotion of nuclear translocation of nuclear factor erythroid 2-related factor 2 (Nrf2) enhances the antioxidant cellular defense by induction of the transcription of antioxidant and cytoprotective genes [96]. Interestingly, it has been reported that activation of HO-1 improves learning and memory function [97]. Moreover, HO-1 is also involved in the inflammatory response. Its upregulation inhibits iNOS and COX-2 and decreases the production of pro-inflammatory cytokines [98]. The direct antioxidant effect of hesperidin and hesperetin involves interaction with ROS via hydrogen donation to free radicals and therefore termination of radical chain reactions. For this activity, the presence of the 3′-hydroxy,4′-o-methoxy system is vital [99]. Owing to antioxidant activity, hesperidin and hesperetin can have a prophylactic effect or suppress the progression of neurodegenerative conditions. 

In AD, the cholinergic neurons are particularly recognized as a region where pathology happens. This group of neurons is involved in many cognitive functions such as learning, memory, attention, and thinking abilities. The main neurotransmitter, taking part in cholinergic signaling and communication between neurons, is acetylcholine (Ach) [100,101]. The loss in cholinergic system function leads to observed symptoms of dementia [102]. Taking into account mentioned factors, regulation of Ach levels in the brain is a site of action which should be considered in the development of therapies. The current AD treatment protocol is based on the administration of cholinesterases—acetylcholinesterase and butylcholinesterase inhibitors such as donepezil, galantamine, rivastigmine, and tacrine [86]. It provides an increase in Ach quantity in synapses and improves cholinergic signaling by making it impossible to break down Ach by cholinesterases. However, drugs relying on this mechanism of action are recognized as symptom-alleviating drugs [103,104]. They do not impact disease progression, so AChE, and BuChe inhibition should be rather viewed as an additional mode of action than the main one in future therapies, aiming at stopping AD progression.

Inflammation is considered a major contributor to the progression of the pathogenesis of neurodegenerative disorders [105]. In neuroinflammation, a significant role is played by microglial cells and proinflammatory mediators [106]. Microglial cells are essential for the protection of the nervous system from pathogens and the promotion of the immune response. However, in the case of neurodegenerative diseases, activated microglia cause the production and release of pro-inflammatory mediators such as nitric oxide (NO), interleukin (IL)-1β, IL-6, and tumor necrosis factor (TNF-α) [107,108]. Their excessive generation may trigger the degeneration of neurons. NO can promote inflammation. Its levels are controlled by iNOS expression [109]. The overexpression of pro-inflammatory cytokines is viewed as a considerable neuroinflammation inducer. TNF-α may ignite cytotoxic cascades and apoptotic pathways of cellular death. Additionally, it has been reported that TNF-α affects learning and memory by interfering with synaptic plasticity, which has an impact on synaptic transmission [110].

Nuclear factor κ-light-chain-enhancer of activated B cells (NF-κB) transcription factor is well known to be engaged in the neuroinflammatory response. It regulates the expression of various genes, including pro-inflammatory mediators such as enzymes (COX, LOX, iNOS) and cytokines (IL-1, IL-6, TNF-α) [111]. In the case of neurodegenerative conditions, the increased activation of NF-κB is observed. It results in the induction of inflammation, which causes the production of neurotoxicity by the generation of ROS, leading to neuronal death [112,113]. NF-κB signaling pathway can be activated by toll-like receptor (TLR) and RAGE. The activation of TLR stimulates defense-signaling pathways as a response to injury or non-physiological cell death initiators [114]. RAGE expression is said to be enhanced in AD patients. Moreover, RAGE can interact with β-amyloid, which activates its signaling [63].

The appearance of β-amyloid plaques is a characteristic feature of AD. β-amyloid proteins are proteolytic elements of the transmembrane amyloid precursor protein (APP) [115]. One of the consequences of aggregation of this protein is the disruption of neurotrophic growth factors metabolism, which is important for cholinergic neurons survival [116,117].

APP is a glycoprotein with a receptor-like structure and is essential in neurite sprouting, branching, and elongation [118]. The metabolism of this cellular element can go in two ways named non-amyloidogenic and amyloidogenic pathways. The first one prevents β-amyloid generation and therefore it can be described as a desirable one. Here, the APP is processed by the α-secretase enzyme, which results in the production of soluble amyloid precursor protein-α (sAPPα). On the other hand, an amyloidogenic pathway is associated with the participation of the β-secretase enzyme (BACE-1) in APP processing, obtaining soluble amyloid precursor protein-β (sAPPβ). Both sAPPα and sAPPβ are further converted by γ-secretase, where sAPPα provides molecules with no pathogenic potential, whereas the transformation of sAPPβ is linked to the generation of β-amyloid peptide species, which are the main constituents of β-amyloid plaques [119,120]. Potential inhibition of BACE-1 and enhancement of α-secretase activity could result in the decreased gathering of neurotoxic β-amyloid plaques and prevent the occurrence of negative effects of their presence in neurons [121].

Aggregation of β-amyloid induces apoptosis by activating caspase-mediated cell signaling pathways [122]. It also attenuates membrane glutamate transporters and generates oxidative stress [123]. The presence of β-amyloid activates the glycogen synthase kinase-3β (GSK-3β). It contributes to the impairment of cognitive functions and promotes apoptosis, which entails neuronal death [124,125]. Additionally, deposition of β-amyloid plaques leads to upregulation of NMDA (N-metyl-D-aspargic acid) receptors, which are involved in the excitotoxicity phenomenon [126]. Excitotoxicity is a major factor contributing to neurodegenerative disorders’ progression. It engages the overstimulation of NMDA receptors, which in turn causes an excessive entrance of calcium ions to intracellular space. Calcium overload ignites cellular signaling cascades, resulting in mitochondrial depolarization, enhanced ROS, and NO production, and further triggering apoptotic pathways and the death of cells [127].

Neurodegenerative diseases are characterized by neuronal loss, which is connected to increased apoptosis occurrence. In the apoptotic process, the balance of Bcl-2/Bax plays an important role. Bax protein can be described as a proapoptotic one, whereas Bcl-2 is an anti-apoptotic one [128]. Both of these factors are involved in the modulation of caspase-3-mediated apoptosis [129]. The growth in proapoptotic mediators such as Bax promotes mitochondrial production of ROS and contributes to neuronal death. Overexpression of Bax triggers the release of cyt c from mitochondria, which is linked to activation of the caspase-3 and -9 pathways, resulting in apoptosis [130]. Additionally, neuronal death can be induced by c-Jun N-terminal kinase (JNK), a stress kinase, ignited by inflammatory mediators and oxidative stress [131].

Another interesting mode of action is the potential beneficial effect on the brain-derived neurotrophic factor (BDNF), a neurotrophin engaged in the growth, differentiation, and survival of the neurons. It plays an essential role in neurogenesis, synaptic plasticity, and memory [132]. In addition, it has been reported that BDNF level is decreased in the brain of patients suffering from AD [133]. Taking into account the importance of BDNF in neuronal function, it seems that the improvement in its levels in the brain is a promising strategy in the treatment of neurodegenerative conditions. It could reverse the negative changes in neuron function caused by pathologic processes.

Apart from beneficial effects in neurodegenerative diseases, described polyphenols might be useful in mood disorders [134]. It is said that one of the major factors contributing to the progression of depression is oxidative stress. Excessive production of ROS leads to increased lipid peroxidation, which causes the destruction of membrane phospholipids, therefore, affecting serotonergic and catecholaminergic receptor functions. It is stated that antioxidants may provide a synergistic antidepressant-like effect when co-administrated with conventional antidepressants [135]. There are some studies indicating the advantageous effect of hesperidin in mood disorders. Souza et al. [136] provided evidence of antidepressant effect via interaction with serotonergic 5-HT_1A_ receptors, whereas Donato et al. [137] suggested that the antidepressant-like effect of hesperidin is connected to an increase of the BDNF levels in the hippocampus as well as inhibition of the L-arginine-NO-cGMP pathway.

## 4. Antidiabetic Activity of Hesperidin and Hesperetin

Diabetes mellitus (DM) is a metabolic condition associated with abnormally increased glucose levels in the blood, which is a consequence of the scarce production or action of insulin. As a result, hyperglycemia occurs [138,139]. DM is becoming a growing problem for world health. There is a prognosis indicating that in 2017 about 425 million people worldwide suffered from this disorder, while in 2045 the morbidity will rise to 629 million worldwide [140]. Considering the increasing occurrence of this condition, there is an urgent need to develop effective therapies.

Hesperidin and its aglycone may be a solution to this struggle since they may act via different modes of action. They show antioxidant, anti-inflammatory, and glucose-regulating properties. Moreover, they affect the production of AGE, which is involved in condition progression and plays a crucial role in the development of complications of DM. The pieces of evidence for the antidiabetic activity of hesperidin and hesperetin are collected in Table 3.

### The Importance of Hesperidin and Hesperetin Modes of Action in DM

Oxidative stress is a major factor contributing to DM pathogenesis. It affects two mechanisms, which are improperly working in this condition—insulin secretion and insulin action [161,162]. Hyperglycemia, occurring in DM promotes oxidative stress through the generation of ROS and suppression of the antioxidant defense systems [163]. Increased production of ROS levels leads to DNA damage and activation of DNA-repairing enzymes. Among others, it results in the accumulation of intermediate products of glucose oxidation, which activates numerous pro-oxidative processes [164]. Glucose oxidation is a physiological process enabling energy production from glucose [165]. However, in hyperglycemia conditions, this process remarkably enhances and generates ROS that exceeds the cellular antioxidant defense systems [166].

Oxidative stress affects insulin action via several mechanisms. It activates uncoupling protein-2 and therefore decreases ATP/ADP ratio, leading to inhibition of insulin secretion cascade depending on ATP [167]. When glucose levels go up in the blood, glucose is uptaken by the β islet cells via GLUT2 transporter, triggering insulin secretion [164]. The entrance of glucose into the β-cell activates glucokinase and glucose-6-phosphate production, resulting in ATP generation [139]. When a high level of ATP in the cell is reached, there is a shutdown of ATP-sensitive potassium channels and sodium influx at the same time. This phenomenon provides depolarization of the membrane and opening of voltage-dependent T-type calcium and sodium channels [168,169]. Increased intracellular calcium concentration promotes the fusion of granules containing insulin with membrane and the release of insulin into the bloodstream [170].

Oxidative stress also impairs the insulin-signaling pathway by affecting PI3-kinase and MAPK [171]. In normal conditions, these pathways are involved in the translation of insulin receptor-generated signals into physiological action, such as promotion of using glucose for protein, lipid, and glycogen synthesis [172] and stimulation of GLUT4 glucose transport, engaged in the uptake of glucose from the bloodstream to peripheral tissues [173]. However, oxidative stress acts as a deactivator of these pathways by enhancing the activity of phosphatases, for instance, protein-tyrosine phosphatase 1B (PTP-1B), and as a consequence inhibiting insulin-receptor signal transduction [174]. Interestingly, the insulin-receptor cascade is also disturbed by oxidative stress. In insulin signaling, the insulin receptor substrate-1 and phosphatidylinositol-3 kinase are key players [175]. In oxidative stress-mediated NO production, there is a decrease in intracellular ATP levels and degradation of insulin signaling components by caspase-3-activated apoptosis [176]. Moreover, oxidative stress may affect insulin gene expression by activating the JNK pathway [177]. Impairment of insulin signaling leads to insulin resistance, which is a pathological condition, related to excess secretion of insulin as a compensation mechanism to maintain the stability of glucose level in the blood since a suitable response to increased glucose is not observed [178].

Owing to antioxidant activity, hesperidin and hesperetin could potentially combat disturbances caused by oxidative stress and therefore reverse or have a protective effect on cells, especially β-cells of the pancreatic islet.

Hyperglycemia is a triggering factor for AGE formation, which is generated by non-enzymatic glycation of free amino groups of proteins. Glycation inactivates enzymes involved in an anti-oxidant defense and indirectly promotes ROS production. On the other hand, increased ROS generation increases AGE levels [141]. There are pieces of evidence suggesting the involvement of AGEs in β-cells damage. They may mediate β-cells toxicity by inhibiting cytochrome-c oxidase and reducing ATP production, therefore interfering with insulin secretion. In addition, AGEs can trigger immune responses causing inflammation to occur, which may lead to apoptosis [179]. The presence of AGE is linked to the occurrence of diabetes complications such as retinopathy [180], nephropathy [181], and cardiovascular complications [182]. An interesting mode of action to combat AGE formation can be the enhancement of Glo-1 enzymatic activity. This enzyme is engaged in the clearance of methylglyoxal, a precursor of AGE [160]. Overexpression of Glo-1 decreases the hyperglycemia-mediated level of AGEs and suppresses inflammation [183]. By affecting AGE production, hesperidin and hesperetin can serve a beneficial effect on diabetes pathogenesis and the development of its complications.

To strengthen the insulin response in cells, the so-called insulin sensitizers can be used. This class of drugs increases insulin sensitivity by acting on intracellular targets such as adenosine 5′-monophosphate-activated protein kinase (AMPK) and peroxisome proliferator-activated receptor gamma (PPAR-γ) [184]. AMPK activation stimulates the energy-generating and inhibits the energy-consuming pathways. Its activation promotes glucose uptake and fatty acids oxidation as well as affects food intake, while in the pancreas it decreases insulin secretion. On the other hand, AMPK activation inhibits gluconeogenesis, fatty acids and cholesterol synthesis, and lipolysis [185]. When it comes to PPAR-γ, it is a transcription factor, which induces the expression of genes engaged in the regulation of glucose homeostasis and lipid metabolism. Its activation enhances the differentiation of fibroblast into adipocytes and increases the gene expression of GLUT4, lipoprotein lipase, and insulin receptor substrates in peripheral tissues. Mentioned actions translate into better insulin sensitivity [186]. As an insulin sensitizer, SIRT-1 (silent mating type information regulation 2 homolog) (*S. cerevisiae*) can be viewed. It is a histone deacetylase, which serves several important roles in cell function. As far as DM is considered, SIRT-1 increases insulin signaling and insulin release and prevents insulin resistance through fat mobilization, mTOR signaling, and inflammation control [187].

An interesting mode of action, connected to insulin secretion, is a dipeptidyl peptidase-4 (DPP-4), an enzyme acting as an inactivator of incretin hormones. Incretins decrease glucose levels in the bloodstream by stimulating insulin release. By inhibition of DPP-4, incretins are protected from degradation and they may serve a physiological role, contributing to proper glucose levels [188,189]. Suppression of DPP-4 activity can be regarded as another mechanism of hesperidin and hesperetin reduction of glucose levels. 

There is increasing evidence that an inflammatory state contributes to the progression of diabetes [190,191]. Inflammation hampers insulin secretion and insulin signaling. Moreover, the influence of proinflammatory mediators in diabetes pathology has been underlined. For instance, IL-1β was recognized as a cytokine that causes the suppression of insulin secretion and the loss of β-cells viability [192]. In addition, it stimulates the generation of NO in β-cells, which negatively affects β-cell glucose oxidation and reduces ATP production. Since an increased amount of ATP in β-cells stands as a signal to insulin release, this phenomenon results in a smaller quantity of insulin in the bloodstream. The damaging action seems to be selective for β-cells [148]. Another important cytokine is IL-6, increased levels of which lead to a decreased IRS-1 tyrosine phosphorylation and a reduced association between the PI-3 kinase and IRS-1, as a consequence suppressing insulin receptor signaling [193]. TNF-α plays a prominent role in the development of an insulin-resistant state. It affects hepatic glucose production by increasing its level [148] and can suppress insulin secretion [194]. It was also highlighted that TNF-α hinders insulin signaling. In addition, this proinflammatory mediator inhibits the GLUT-4 expression; therefore, it affects the ability of peripheral tissue to intake glucose from blood [195]. Bearing in mind the essential role of inflammation in DM development and progression, the anti-inflammatory actions of hesperidin and hesperetin are useful.

It is believed that β-cells loss related to apoptosis is a crucial factor in the onset and progression of DM, causing insulin deficiency [196], and hence anti-apoptotic activity may be a game-changer in the pathology of this condition. High glucose level is a triggering factor for ROS generation. Oxidative stress induces the reduction of mitochondrial membrane potential, damage to the mitochondrial membrane, and liberation of cytochrome c, which promotes the activation of caspase-mediated cell death [197]. One of the possible mechanisms to suppress apoptotic death is to influence the level of apoptosis mediators such as Bcl-2 (anti-apoptotic protein) and Bax (pro-apoptotic protein). Shifting the ratio of Bcl-2/Bax in favor of Bcl-2 can serve a protective effect regarding cell survival [198]. Owing to this phenomenon, there can be a blockage of caspase activation. Moreover, impact on the MAPK pathway can influence p38 and JNK-mediated activation of cellular death; therefore, affecting this cascade can keep the cells safe [199]. Thanks to the anti-apoptotic activity of hesperidin and hesperetin, they may act as protectants of cells, contributing to the suppression of DM progression.

Interestingly, there are reports of the potential synergistic effect of hesperetin and trans-resveratrol combination. Combined intake of these two induced the expression of glyoxalase 1, fighting with the gathering of methylglyoxal and protein glycation, and therefore causing the reversal of insulin resistance [200]. It is worth noting that the mentioned polyphenols individually were ineffective, and the positive observation regarding insulin resistance was observed only when co-administrated [201].

Regardless of its great potential in studies, hesperidin seems not to shine in human clinical trials, when it comes to its antidiabetic activity. Shams-Rad et al. performed a meta-analysis of randomized controlled clinical trials concerning hesperidin, in which they claimed that supplementation of this polyphenol might not be a considerable agent to improve glucose control [202]. However, this fact could be connected to the poor bioavailability of this molecule.

## 5. Conclusions

To sum up, hesperidin and hesperetin seem to be very attractive compounds in terms of pro-health activity. However, poor bioavailability stands out as an obstacle, which should be overcome to know their true potential. Hesperidin seems to be of particular interest due to its relatively low production prices. Still, in this case, attention should also be paid to the great importance of intestinal flora. Therefore, in human studies, parallel supplementation with probiotics should be carried out to maximize the potential of the formulation. Considering the studies presented, there is significant potential for hesperidin and its aglycone in the fight against chronic diseases such as neurodegenerative diseases and diabetes. Hence they may be a game-changer in the treatment of these diseases when their bioavailability is improved using various delivery systems.

## Figures and Tables

**Table 1:** Collected attempts to increase the bioavailability of hesperidin and hesperetin.

Hesperidin		
**Technique**	**Observations**	**Reference**
Hesperidin–chitosan complexes	The enhancement of solubility by 1.6-, 2.7-, and 3.8-fold and visible correlation between improved solubility and antioxidant activity. The greater the solubility improvement was, the better antioxidant activity reported	[16]
Inclusion complex of hesperidin with HP-β-CD	Obtaining the complex translated into increased solubility by 95-fold with respect to unmodified compound	[17]
Solid lipid nanoparticles loaded with Hesperidin	The increase of solubility by 20-fold. Impact on apparent permeability, leading to enhancement nearly by 5-fold. After oral administration, the overall bioavailability increased by 4.5-times in the study performed in a rat model. The obtained system affected biological activity as well, providing attenuation of Doxorubicin-induced cardiotoxicity and oxidative stress	[34]
Amorphous systems of Hesperidin with mesoporous material	Significant improvement in solubility by 51-fold for the best system and an impact on dissolution rate, better dissolution behavior in terms of apparent solubility	[35]
Nanoparticles of Hesperidin loaded by PLGA-Poloxamer 407	In in vitro release profiles, sustained and slow release, and higher apparent solubility were observed. This modification provided stronger inhibitory activity on the breast cancer cells	[36]
Hesperidin-β-CD inclusion complexes	The systems showed better behavior in dissolution studies and also demonstrated an enhancement of antibacterial and antioxidant activity compared with unmodified hesperidin	[37]
Inclusion complexes of Hesperidin with HP-β-CD	The obtained complexes showed an improvement in dissolution rate, and antioxidant as well as antimicrobial activity	[38]
A Solid self-microemulsifying system with Hesperidin composing of Maisine CC, Tween 80 and PEG 400	Significantly better dissolution rate profiles than that of free hesperidin, which enabled the release of almost all polyphenol from the system (>98%) after 60 min. Moreover, formulation showed better therapeutic activity for the management of diabetes mellitus in vivo	[39]
Solid nanocrystals	In the solubility studies, the system provided enhancement in solubility by 4.8-fold with respect to pure compound, faster dissolution, and higher apparent solubility	[40]
Inclusion system of Hesperidin with octenyl succinic anhydride modified sweet potato starch	The increase in solubility by 6.52-fold in the optimal conditions	[41]
Nanocrystals by combining Hesperidin with HPMC E5 and Poloxamer 188	The systems enhanced the solubility by 5-times as well as the drug dissolution rate. The systems were characterized by comparable antioxidant activity with regard to pure compound.	[42]
Hesperidin-PEG 6000 complex	Enhancement of solubility by 21-fold.	[43]
**Hesperetin**		
Cocrystals with different excipients such as caffeine, nicotinamide and picolinic acid	It translated into about 5-times better solubility as compared with pure substance. The parachute effect was observed in dissolution rate studies. Moreover, significant improvements in biological activity and pharmacokinetic profile were noticed.	[44]
Eutectic mixtures	In dissolution studies, the increase of apparent solubility was evident and reached about 3-times higher than the pure compound. The biological models revealed a direct impact of solubility on antioxidant and antihemolytic activity	[45]
Complexes of Hesperetin with β-CD and HP-β-CD	Higher solubility by 25-fold for β-CD and 467-fold for HP-β-CD complexes.	[46]
Nanocrystals	Significant enhancement in dissolution rate and apparent solubility was reported. In dissolution rate studies, authors reported the spring effect, leading to a dramatic increase in solubility in a short time from the beginning. However, the amount of dissolved substance decreased over time, and thus the parachute effect was not observed.	[47]
The systems of Hesperetin with Mg- or Ag-modified SBA-16 carriers	In dissolution studies, higher apparent solubility and dissolution velocity were reported. However, the total drug release was unnoticed.	[48]
Nanoemulsion	The authors reported 5.67-fold higher oral bioavailability	[49]
Nanoparticles composed of Hesperetin and Eudragit E 100	Systems were characterized by sustained release with a pattern of initial rapid release of about 30% of the drug in the first 8 h, followed by a slow and continuous release of approximately 82% drug release in the next 24 h.	[50]
Self-assembling rebaudioside A nanomicelles with hesperetin	A drug release study revealed that prepared systems considerably increased apparent solubility and provided sustained release of the compound, reaching almost 81% at 24 h time point. This approach had a positive impact on the biological activity of hesperidin with respect to anticancer efficacy.	[51]
Formulations of hesperetin-D-alpha-tocopheryl polyethylene glycol 1000 succinate micelles and hesperetin-phosphatidylcholine complexes	The micelles formation was connected to an increase of solubility of 21.5-fold, whereas phosphatidylcholine complexes by 20.7-fold. Moreover, the solubility enhancement translated into a 4.2-fold boost in antioxidant activity for micelles and 3.9-fold for complexes. A significant improvement in bioavailability was also reported. The AUC increased by 16.2-fold for micelles formulation, whereas for complexes it was 18.0-fold.	[52]
Hesperetin complexes with β-CD and methylated-β-CD	The complexation caused an increase in apparent solubility and improved the dissolution profile. It also helped to increase the anti-inflammatory activity by reducing IL-6 secretion from LPS-stimulated macrophages.	[53]
Hesperetin-PLGA nanoparticles	Sustained release from formulation, which enabled a constant, slow-release within 7 days. Enhancement in the cytotoxic activity of prepared delivery system as compared with free compound.	[54]
Biocompatible gold nanoparticles of hesperetin	Sustained release of hesperetin from nanoparticles and increased cytotoxicity on cancer cells.	[55]
Chitosan-based nanoparticles	Sustained release of hesperetin and enhanced anticancer activity by an increase of inhibitory effect on colon cancer cell growth by 6-fold.	[56]

**Table 2:** Collected studies of pure compounds suggesting neuroprotective activity of hesperidin and hesperetin.

Hesperidin		
**Model**	**Observations/proposed mechanism**	**Reference**
Human neuroblastoma SK-N-SH cells	- Maintenance of mitochondrial membrane potential - Antioxidant—increase in glutathione, SOD, GSH-Px levels - Antiapoptotic—downregulation of Bax, caspase-3, 9; upregulation of Bcl-2	[58]
Neuro-2A cells	- Inhibition of β-amyloid-induced autophagy - Improved glucose utilization	[59]
In silico In vitro	- Inhibition of cholinoesterases—acetylcholinesterase (AChE), butyrylcholinesterase (BChE) - Inhibition of β-secretase 1 (BACE 1)	[60]
female C57 BL/6 mice	- Antidepressant-like effect - Improvement of cognitive performance and spatial memory - Antioxidant—increase in antioxidant enzymes activity and glutathione levels	[61]
Male Albino Wistar rats	- Decrease in AChE activity - Improved learning and memory - Suppression of APP, β-amyloid, β-, γ-secretases expression	[62]
Male APP/PS1 mice	- Improvement in learning and memory - Anti-inflammatory and anti-oxidant via activation of Akt/Nrf2 and inhibition of RAGE/NF-κB signaling pathways	[63]
In silico In vitro	- Anti-amyloidogenic—BACE-1 inhibition - Antioxidant	[64]
APPswe/PS1dE9 mice	- Improvement in learning and memory - Amelioration of recognition memory - Antioxidant—an increase of antioxidative defense; decrease in GKS-3β activity	[65]
Adult male C57BL/6 mice	- Amelioration of motor dysfunction - Anti-inflammatory—suppression of microglia activation; inhibition of COX-2 and attenuation of inflammatory cytokines—IL-1β, IL-4, IL-6, IL-10, TNF-α release	[66]
Male Wistar rats	- Anti-apoptotic—a decrease of Bcl-2 and increase of Bax expression - Amelioration of learning and memory - Antioxidant—increase in glutathione levels; enhancement of antioxidant enzymes activity—SOD, CAT, GPx	[67]
male transgenic APP/PS1–21 mice	- Decrease in microglial activation - Decrease in TGF-1β expression - Anti-amyloidogenic—attenuation in β-amyloid depositions accumulation and APP expression	[68]
Swiss male albino mice	- Attenuation of AChE activity - Anti-inflammatory—inhibition of NF-κB pathway and the release of COX-2 and iNOS - Inhibition of astrocytes activation - Improved memory consolidation	[69]
**Hesperetin**		
adult male mice (C57BL/6N, wild type) HT22 cells	- Decrease in oxidative stress (via increase of Nrf2 HO-1 expression) - Anti-neuroinflammatory effect (reversion of β-amyloid-induced activation of astrocytes and microglia; decrease in TLR4, NF-κB expression) - Anti-apoptotic (downregulation of proapoptotic markers—Bax, Caspase-3, PARP-1; up-regulation of anti-apoptotic marker—Bcl-2) - Regulation of synaptic markers—increase in Syntaxin, SNAP-25, PSD-95, Syp, and SNAP-23 levels - Alleviation of short-term memory dysfunction	[70]
PC12 cells	- Antioxidant—induction of PKA, PI-3K, PGC-1α, and seladin-1 via ER- and TrkA-meditated pathways	[71]
Wistar rats	- Improvement in learning and recognition memory - Antioxidant (increase in glutathione and CAT, SOD, GR_X_, and GP_X_ levels and decrease of lipid peroxidation)	[72]
PC12 cells	- Decrease in Ca^2+^ level - Antioxidantan increase in CAT, GSH-Px, and GRx levels - Decrease in caspase-3 activity - Maintenance of mitochondrial membrane potential - Decrease in DNA damage	[73]
Neuro-2A cells	- Inhibition of β-amyloid-induced autophagy - Improved glucose utilization	[59]
In silico In vitro	- Inhibition of cholinesterases—acetylcholinesterase (AChE), butyrylcholinesterase (BChE) - Inhibition of β-secretase 1 (BACE 1)	[60]
ICR female mice	- Antioxidant—activation of antioxidant enzymes—CAT, SOD	[74]
Male albino Wistar rats	- Decrease in AChE activity - Maintenance of mitochondrial membrane potential - Antiapoptotic—decrease in Bax, caspase-3, 9 levels and increase in Bcl2 - Antioxidant—increase in CAT, SOD, Gpx, GST activity	[75]
Male C57BL/6 N mice	- Antioxidant—decreased production of ROS and increased antioxidant proteins Nrf2, HO-1 levels - Anti-inflammatory—decreased expression of proinflammatory cytokines—TNF-α, IL-1β, p-NF-κB - Antiapoptotic—decreased expression of p-JNK, Bax, and caspase-3 increased expression of Bcl-2 - Enhanced synaptic integrity, cognition, and memory process	[76]
Male adult Wistar rats PD	- Improved motor functions - Attenuation of apoptosis by increased Bcl2 expression - Attenuation of astrogliosis by a decrease in GFAP levels - Decreased neuroinflammation by reduction of NF-κB levels	[77]
Cortical cells	- Inhibition of NMDA-induced excitotoxicity caused by the excess of glutamate - Protection against β-amyloid-induced neuronal damage	[78]
C57/BL6 male mice BV-2 microglial cells	- Inhibition of astrocyte and microglial activation - Anti-inflammatory—attenuation of production of iNOS, NO, IL-6, IL-1β	[79]
SH-SY5Y cells	- Attenuation of apoptosis—decrease in caspase-3, -9 expression - Antioxidant—increased levels of GSH, SOD, and expression of NRF2 and HO-1	[80]
Male albino mice	- Improved spatial learning and reference memory - Maintenance of cholinergic neurotransmission - Inhibition of AChE activity - Antioxidant—increase in SOD and GSH levels - Increased BDNF levels	[81]
RAW 264.7 Cells	- Anti-inflammatory effect concerning inhibition of NF-κB and activation of Nrf2/HO-1—suppression of proinflammatory cytokines (TNF-α, IL-6, IL1β) and pro-inflammatory enzymes (iNOS, COX-2) expression	[82]

**Table 3:** Collected studies of pure compounds suggesting antidiabetic activity of hesperidin and hesperetin.

Hesperidin		
**Model**	**Observations/proposed mechanism**	**Reference**
Rat skeletal muscle cell lines, L6 myoblasts	- Antioxidant—free radicals scavenging; increase in glutathione levels - Increased glucose uptake—up-regulation of GLUT-4 receptors and down-regulation of PI3 kinase	[141]
Male Sprague Dawley rats	- α-glucosidase inhibition	[142]
In vitro In silico	- Dipeptidyl peptidase-4 inhibition	[143]
In vitro—Caco-2/TC7 cells and *Xenopus laevis* oocytes In vivo—human	- Decrease in sugar absorption rate by inhibition of GLUT 2 and GLUT 5 transporters	[144]
Male Sprague Dawley rats	- Regulation of glycolysis and gluconeogenesis—induction of glucokinase and decrease in glucose-6-phosphatase and phosphoenolpyruvate carboxykinase activity - Improved insulin sensitivity by activating the IR/PDK1 pathway - Improved glucose uptake	[145]
Male Wistar rats	- Up-regulation of GLUT 4 translocation - Anti-apoptotic effect—increase in antiapoptotic Bcl-2 protein and decrease in pro-apoptotic protein Bax levels - Increased PPAR-γ expression	[146]
In silico In vitro—pancreas of male BALB/c mice	- Glucose-dependent insulinotropic effect by PKA-dependent mode of action	[147]
White male albino rats	- Reduction in oxidative stress—enhanced antioxidant enzymes (CAT, GPx, GR, SOD) levels - Anti-inflammatory—suppression in the production of pro-inflammatory cytokines—TNF-α, IL-6 - Decreased glucose, glycosylated hemoglobin, and increased insulin plasma levels	[148]
Male Wistar albino rats	- Reduction in insulin, total cholesterol, triglyceride, low-density lipoprotein cholesterol serum levels - Improved histological structure - Anti-inflammatory—decreased pro-inflammatory cytokines (TNF-α, IL-6) levels	[149]
Retinal ganglion cell 5 (RGC-5) cells	- Antioxidant—enhancement in SOD, GPx, CAT activities - Anti-apoptotic—stabilization of mitochondrial membrane potential, inhibition of caspase-3, -9, and Bax expression, enhancement in Bcl-2 expression, suppression in pro-apoptotic p38 and JNK MAPK pathways activation	[150]
Male albino rats	- Decrease in fasting blood glucose and glycosylated hemoglobin levels - Increase in insulin secretion—protective action on β-cells and stimulatory effect on the insulin secretory response of islets of the pancreas - Decrease in gluconeogenic enzymes - Anti-hyperglycemic—increased GLUT 4 expression	[151]
**Hesperetin**		
Rat skeletal muscle cell lines, L6 myoblasts	- Antioxidant—free radicals scavenging; increase in glutathione levels - Increased glucose uptake—up-regulation of GLUT-4 receptors and down-regulation of PI3 kinase	[141]
Male albino Wistar rats	- Reduction in glucose plasma and increase in insulin levels similar to glibenclamide - Recuperation of pancreatic β-cells - Improvement in glucokinase activity and glucose-6-phosphate dehydrogenase - Inhibition of hepatic gluconeogenesis—decrease in the level of gluconeogenic enzymes—glucose-6-phosphatase and fructose-1,6-bisphosphatase - Increased glycogen content in hepatocytes - Antioxidant—increase in activity of enzymic antioxidants - Anti-hyperlipidemic effect—enhanced insulin secretion, which led to a reduction in cholesterol synthesis and due to the ability of hesperetin to bind bile acids, which resulted in a decrease in the cholesterol absorption - Protective effect on hepatic damage - Renoprotective effect	[152]
Wistar rats	- Attenuation of gluconeogenesis by inhibition of mitochondrial pyruvate carrier, uncoupling of mitochondrial oxidative phosphorylation, inhibition of mitochondrial respiratory chain at Complex I, and deviation of NADH supply for gluconeogenesis and mitochondria due to a prooxidant action, deviation of glucose 6-phosphate for glucuronidation reactions	[153]
Adult male Wistar albino rats	- Reduction of plasma glucose because of the increased release of insulin from the existing β-cells and/or regenerated β-cells of the pancreas, restored insulin sensitivity or inhibition of intestinal absorption of glucose, or enhanced the utilization of glucose by peripheral tissues - Improved lipid profile - Improved pancreatic islets’ morphology	[154]
Male mice	- Anti-hyperglycemic—increased insulin production and reduced blood glucose levels	[155]
Wistar rats	- Anti-hyperglycemic—decrease in glucose levels - Antioxidant—increase in antioxidant enzymes—SOD, CAT, GSH, GPx activity - Anti-inflammatory—decrease in pro-inflammatory cytokines levels—TNF-α, IL-17 - Anti-apoptotic—suppression of caspase-3 and maintenance of mitochondrial membrane potential	[156]
In vitro In silico	- α-glucosidase inhibition	[157]
In vitro In silico	- Dipeptidyl peptidase-4 inhibition	[143]
HepG2 cells	- Increase in protein level and direct activation of SIRT1, which was accompanied by induction of AMPK phosphorylation	[158]
RAW264.7 cells	- Inhibitory effect on oxidative stress and inflammation induced by AGEs	[159]
Male Sprague Dawley rats	- Up-regulation and Increased Glo-1 enzymatic activity - Anti-inflammatory—decreased level of pro-inflammatory cytokines (IL-1β, TNF-α) - Enhancement of Nrf2/ARE pathway	[160]

## Data Availability

Not applicable.
